# Metabolic Signatures of Triatomine Vectors of *Trypanosoma cruzi* Unveiled by Metabolomics

**DOI:** 10.1371/journal.pone.0077283

**Published:** 2013-10-30

**Authors:** Luis Caetano M. Antunes, Jun Han, Jingxi Pan, Carlos J. C. Moreira, Patrícia Azambuja, Christoph H. Borchers, Nicolas Carels

**Affiliations:** 1 Michael Smith Laboratories, The University of British Columbia, Vancouver, Canada; 2 National Institute for Science and Technology on Innovation on Neglected Diseases (INCT/IDN, CNPq), Centro de Desenvolvimento Tecnológico em Saúde, Fundação Oswaldo Cruz, Rio de Janeiro, Brazil; 3 Escola Nacional de Saúde Pública Sergio Arouca, Fundação Oswaldo Cruz, Rio de Janeiro, Brazil; 4 University of Victoria – Genome BC Proteomics Centre, University of Victoria, Victoria, Canada; 5 Laboratório de Doenças Parasitárias, Instituto Oswaldo Cruz, Fundação Oswaldo Cruz, Rio de Janeiro, Brazil; 6 National Institute for Molecular Entomology (INCT/ME, CNPq), Laboratório de Bioquímica e Fisiologia de Insetos, Instituto Oswaldo Cruz, Fundação Oswaldo Cruz, Rio de Janeiro, Brazil; 7 Laboratório de Genômica Funcional e Bioinformática, Instituto Oswaldo Cruz, Fundação Oswaldo Cruz, Rio de Janeiro, Brazil; Universidade Federal do Rio de Janeiro, Brazil

## Abstract

Chagas disease is a trypanosomiasis whose causative agent is the protozoan parasite *Trypanosoma cruzi*, which is transmitted to humans by hematophagous insects known as triatomines and affects a large proportion of South America. The digestive tract of the insect vectors in which *T. cruzi* develops constitutes a dynamic environment that affects the development of the parasite. Thus, we set out to investigate the chemical composition of the triatomine intestinal tract through a metabolomics approach. We performed Direct Infusion Fourier Transform Ion Cyclotron Resonance Mass Spectrometry on fecal samples of three triatomine species (*Rhodnius prolixus*, *Triatoma infestans*, *Panstrongylus megistus*) fed with rabbit blood. We then identified groups of metabolites whose frequencies were either uniform in all species or enriched in each of them. By querying the Human Metabolome Database, we obtained putative identities of the metabolites of interest. We found that a core group of metabolites with uniform frequencies in all species represented approximately 80% of the molecules detected, whereas the other 20% varied among triatomine species. The uniform core was composed of metabolites of various categories, including fatty acids, steroids, glycerolipids, nucleotides, sugars, and others. Nevertheless, the metabolic fingerprint of triatomine feces differs depending on the species considered. The variable core was mainly composed of prenol lipids, amino acids, glycerolipids, steroids, phenols, fatty acids and derivatives, benzoic acid and derivatives, flavonoids, glycerophospholipids, benzopyrans, and quinolines. Triatomine feces constitute a rich and varied chemical medium whose constituents are likely to affect *T. cruzi* development and infectivity. The complexity of the fecal metabolome of triatomines suggests that it may affect triatomine vector competence for specific *T. cruzi* strains. Knowledge of the chemical environment of *T. cruzi* in its invertebrate host is likely to generate new ways to understand the factors influencing parasite proliferation as well as methods to control Chagas disease.

## Introduction

The protozoan parasite *Trypanosoma cruzi* is the causative agent of Chagas disease, an endemic infection in much of Latin America [1], [2], [3]. The disease is spread to humans through hematophagous insect vectors called triatomines, which are members of the *Reduviidae* family and the *Triatominae* subfamily [3] [4]. The dynamics of parasite-vector interactions are very complex [5], [6], [7]; different vector species show distinct geographic distributions, and certain *T. cruzi* strains are associated with particular insect species [4], [8]. Over 100 species of triatomines can act as vectors of Chagas disease in a process that involves several transmission and adaptation steps [3], [4]. All of these factors come into play to determine the distribution and epidemiology of the disease, for which there are still no efficacious treatment options.

The cycle of *T. cruzi* transmission begins when a triatomine ingests the parasite during a blood meal from an infected human or animal. The parasite then passes through the triatomine digestive tract and undergoes a number of morphological differentiations that result in the production and multiplication of epimastigote parasites [9]. During the next blood meal, the insect excretes a number of infective trypomastigote parasites in the stool and urine, and these parasites can enter their new host through the vector's bite or directly through the mucosa. The newly infected host can then serve as a reservoir for further parasite dissemination [3], [4]. During this transmission cycle, the transformations experienced by *T. cruzi* upon entering the insect vector involve several steps (reviewed in [9]).

During its journey in the invertebrate host, *T. cruzi* must survive within the digestive constraints of the triatomine gut. In adult *Rhodnius*, the midgut is divided into two major regions, the stomach (anterior midgut) and intestine (posterior midgut; I), which itself is further divided into anterior (AI; mainly for secretion) and posterior (PI; mainly for absorption) segments. Morphologically, the AI and PI cells from adult *Rhodnius prolixus* and *Triatoma infestans* can be separated on the basis of their shape and corresponding specific post-feeding modifications, suggesting differences in their digestive process [10]. Hemoglobin digestion is initiated in the AI, which is also the major region for the synthesis and secretion of digestive proteinases, such as cathepsins B and D, carboxypeptidase B, and aminopeptidase. Protein digestion takes place only in the AI, where a complex extracellular membrane layer (ECML) that functions as a peritrophic membrane forms over the apical cell surface of microvilli 12–24 hours after feeding [11]. Initial digestion occurs inside the endoperitrophic membrane, intermediate digestion in the ectoperitrophic space, and final digestion at the surface of PI cells by integral microvillar enzymes or enzymes trapped in the glycocalyx [10]. Cathepsin B, cathepsin D and carboxypeptidase B reach maximum activity 6–7 days after feeding [11]. The terminal digestion stage of blood proteins is carried out by an aminopeptidase retained on the microvilli and in the ECML of the intestinal cells [11]. Because enzymatic activity increases in the lumen after feeding, extracellular membrane layer development continues until it separates the intestinal cells from the lumen 6–7 days after feeding. The PI is clearly the major site of nutrient absorption and continues to accumulate sugars until at least 20 days after feeding [10]. Additional hydrolase activity may also derive from obligate and facultative bacterial symbionts that are commonly found in triatomines [12].

Many studies have produced strong evidence that the parasite has an intimate interaction with its invertebrate host environment and that there is a clear coevolution between *T. cruzi* strains and insect vector species; many variables play a role in modulating these interactions [4], [5], [6], [7], [9], [13]. Therefore, the parasite must have strategies to cope with the challenges presented by such environmental variations as well as mechanisms to adapt to and take advantage of them. In this sense, the intestinal environment of the insect host is particularly relevant, and the microbes and chemicals encountered in such an environment are likely to affect the parasite-host interaction and the fate of the association. Indeed, many molecules present in the intestinal tract of triatomine hosts have been shown to affect *T. cruzi* [14]. For instance, the hemolytic factor present in the stomach of *R. prolixus* has the capacity to lyse the epimastigote forms of some *T. cruzi* strains but not others [15]. Lectins can agglutinate some *T. cruzi* strains [15]. Additionally, studies of bacterial strains isolated from the triatomine gut revealed that strains of *Serratia marcescens,* the predominant bacterial species found in *Rhodnius* and *Triatoma* [12], are able to lyse epimastigote forms of *T. cruzi* [16]. Altogether, these observations show that a myriad of compounds found in the triatomine gut have a decisive influence in the infectious process and determine whether *T. cruzi* can successfully establish itself in the vector host, which will subsequently determine its capacity to spread through human populations.

We have recently shown that although the intestinal microbiota of triatomines is lacking in bacterial species diversity, it displays marked specificity with certain bacteria associated with different insect vectors [12]. Understanding the molecular basis of the vector-parasite-commensal bacteria triad may aid in our knowledge of their determinants and the epidemiology of *T. cruzi* infections in humans and in the identification of promising therapeutics. Therefore, we set out to study the chemical environment of the triatomine gut through a powerful, high-throughput metabolomics approach. Our results indicate that metabolic variation among triatomine species is obvious even in laboratory conditions. We describe the identification of uniform and variable cores of metabolites in three different triatomine species. Our results provide a basis for further investigations of the interplay between *T. cruzi* and the triatomine digestive tract and how this may come into play during vector colonization, especially in the hindgut, where parasite metacyclogenesis occurs. Ultimately, understanding metabolic variations may allow the identification of factors regulating parasite growth and assist in the development of anti-parasitic drugs.

## Materials and Methods

### Chemicals

Haloperidol, reserpine, acetonitrile, water, formic acid, and ammonium hydroxide were purchased from Sigma-Aldrich (St. Louis, USA). Electrospray (ES) tuning mix was purchased from Agilent Technologies (Santa Clara, USA).

### Triatomine rearing and sample collection

The triatomine species used in this study are from the insectary of the *Laboratório de Doenças Parasitárias* from the *Instituto Oswaldo Cruz*. These insects were fed weekly on chickens and raised as previously described [17] according to the Ethical Principles in Animal Experimentation approved by the Ethics Committee in Animal Experimentation (CEUA/FIOCRUZ) under protocol number P-54/10-4/LW12/11. The specimens used in these experiments (*Rhodnius prolixus*, *Triatoma infestans*, *Panstrongylus megistus*) were fasted for approximately 15 days and were then fed with defibrinated rabbit blood using an artificial apparatus similar to that described previously [18] according to the Ethical Principles in Animal Experimentation approved by the CEUA/FIOCRUZ under protocol number L-0061/08. Both protocols are from CONCEA/MCT (http://www.cobea.org.br/), which is associated with the *American Association for Animal Science* (AAAS), the *Federation of European Laboratory Animal Science Associations* (FELASA), the *International Council for Animal Science* (ICLAS) and the *Association for Assessment and Accreditation of Laboratory Animal Care International* (AAALAC). The fed and engorged insects were individually placed in plastic tubes adapted to collect feces [19]. When insects defecated, the stools were immediately collected and stored at −20°C. All insects used in this study were at the same developmental stage (fifth instar), and sample collection was limited to up to three hours post feeding.

### Sample preparation

Feces were collected from three *T. infestans*, three *R. prolixus*, and three *P. megistus* individuals, for a total of nine fecal samples of three different triatomine species. Fecal samples were weighed, and 40 mg of material were freeze-dried and used in the subsequent procedures. For metabolic profiling, the dried extracts were dissolved in 400 µL of 50% acetonitrile (in water), sonicated and centrifuged to pellet precipitates. The supernatant was collected and diluted 1∶4 with 50% acetonitrile containing either 0.2% formic acid (for positive-ion mode) or 0.2% ammonium hydroxide (for negative-ion mode) and spiked with known amounts of haloperidol, reserpine, and the ES tuning mix as the internal standards for mass calibration.

### Direct Infusion Fourier Transform Ion Cyclotron Resonance Mass Spectrometry (DI-FT-ICR-MS)

Samples were infused through a syringe pump (KDS Scientific, Holliston, USA) at a flow rate of 2.5 µL per minute into a 12-Tesla Apex-Qe hybrid quadrupole-FT-ICR mass spectrometer (Bruker Daltonics, Billerica, USA) equipped with an Apollo II electrospray ionization source, a quadrupole mass filter, and a hexapole collision cell. Data were recorded in positive- and negative-ion modes with broadband mode detection and an FT acquisition size of 1,024 kilobytes per second within a range of *m/z* 150 to 1,000. Under these settings and following internal mass calibration, a mass resolution of ca. 100,000 (full width at half maximum) at *m/z* 400 and accuracy within 2 ppm or less for all detected components were observed. The other experimental parameters used were as follows: capillary electrospray voltage of 3,600 to 3,750 V, spray shield voltage of 3,300 to 3,450 V, source ion accumulation time of 0.1 s, and collision cell ion accumulation time of 0.2 s. To increase detection sensitivity, mass spectra were acquired from the accumulation of 200 scans per spectrum.

### Data processing

Data analysis was performed essentially as previously described [20], [21], [22], [23]. First, raw mass spectra acquired from each triatomine species were batch-processed using the instrument vendor's data analysis software, DataAnalysis, but with a script written in-house to perform automatic internal mass calibration with the reference masses of the spiked calibration standards. Monoisotopic peaks corresponding to the isotopic pattern distributions were automatically determined, and those with a signal/noise ratio above three were used. Peak intensities were aligned and combined into unique metabolite features from the *m/z* that matched within 2 ppm across all the data. A data matrix of *m/z* versus relative intensity was generated for each sample group and saved for further analysis. These data are shown in Table S1. To identify differences in metabolite composition among the triatomine species, we compared the list of normalized *m/z* peak intensities of each species and filtered it for metabolites that were present in one of the species at a relative frequency that was statistically higher than that of at least one of the others. Those *m/z* showing intensities statistically higher in one triatomine species compared to either of the other two were considered to belong to the *variable* core. The remaining metabolites were considered part of the *uniform* core. Both groups were used for further analyses. Due to the lack of an appropriate database for the analysis of metabolomics work involving triatomines, *m/z* of interest were queried against the Human Metabolome Database (HMDB, http://www.hmdb.ca/). Searches were performed within an *m/z* error of 0.001 Da. Unlikely ion adducts (isopropanol, methanol, dimethyl sulfoxide, formic acid, acetic acid, trifluoroacetic acid, and bromide) were manually removed from the resulting lists, and the HMDB *Class* or *Subclass* annotation was used for the purpose of metabolite category assignment. For metabolic classification, whenever a given metabolite produced multiple hits in the database search, only one assignment among redundant hits from the same metabolic category was considered. A detailed analysis of this strategy compared to the use of multiple assignments revealed that both strategies produced similar results concerning the relative distributions of metabolic categories (data not shown). In this study, we adopted the non-redundant assignment strategy because it offered a convenient means of automation.

### Statistical analysis

Considering one fecal sample of three individual replicates among three triatomine species (*T. infestans*, *R. prolixus*, *P. megistus*), we obtained 2,086 *m/z* peaks by *DI-FT-ICR-MS* (combining results from positive and negative ionizations). As the first step of data analysis, we normalized the absolute *m/z* peak intensities to the total intensity of each sample. Thus, the sum of relative frequencies of metabolites was always 100%, and the sum of their differences among replicate pairs was always zero. Because the distribution of replicate pair differences was normal and centered on zero for each case, we looked for the two tail threshold values between which 95% of data were found. In general terms, metabolites outside of this range were considered to be significantly different between species. A detailed description of the statistical analysis is provided as Methods S1.

## Results

### Statistics of metabolites in triatomine feces

**Figure 1:**
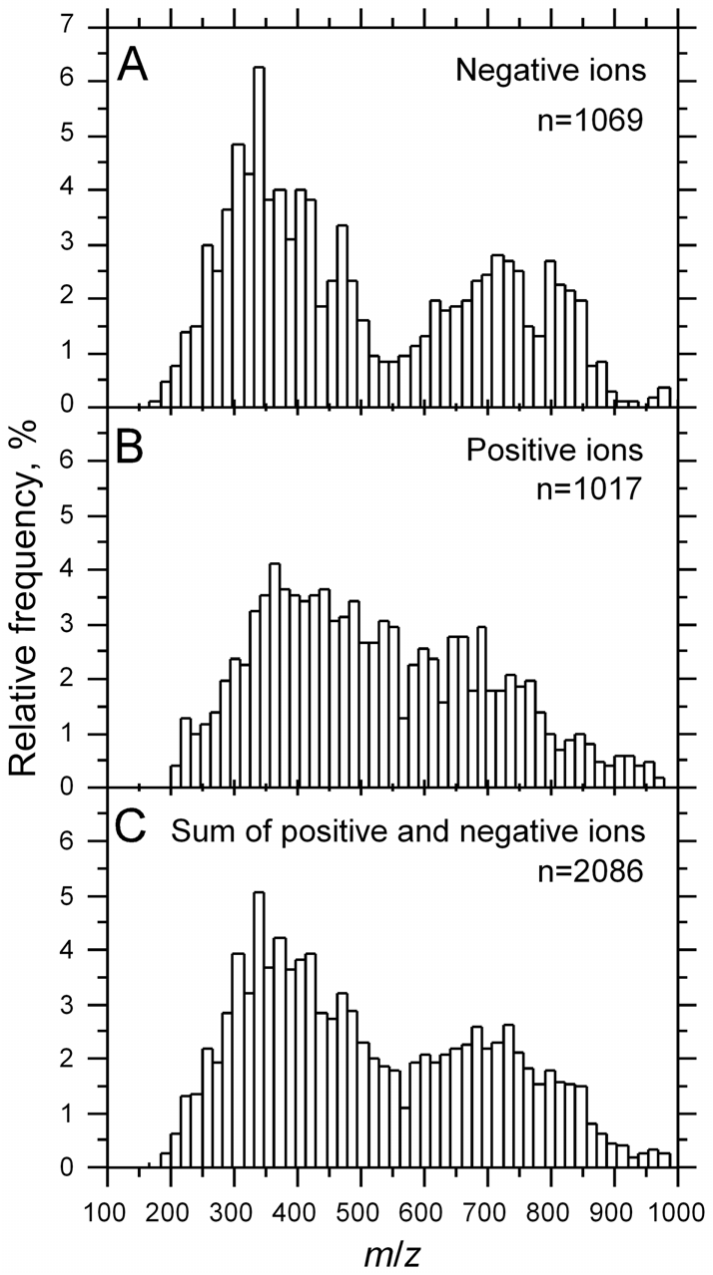
Distribution of metabolites in triatomine feces. A: Negative ions, B: Positive ions, C: Sum of negative and positive ions.

To study the metabolic composition of the triatomine gut, we performed DI-FT-ICR-MS on fecal samples from *Triatoma infestans*, *Rhodnius prolixus* and *Panstrongylus megistus*. DI-FT-ICR-MS allowed us to detect a combined total of 2,086 metabolite features from all species, 1,069 in negative ionization mode and 1,017 in positive ionization mode. The distribution of metabolites in the feces of triatomines is bimodal and asymmetrical, with the left mode representing negative ions (Figure 1A), and a shoulder replacing the mode on the right for positive ions (Figure 1B). As a result, the distribution of the sum of negative and positive ions is bimodal as well (Figure 1C).

**Figure 2:**
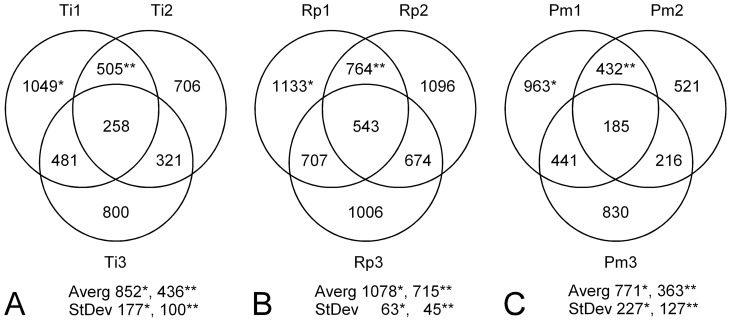
Distribution of metabolites among triatomines. Venn diagrams represent metabolite frequencies (numbers in circles) and their share among replicates (circles) for each triatomine species (A: *T. infestans* or Ti, B: *R. prolixus* or Rp, C: *P. megistus* or Pm). For example, the sample size of replicate number 1 of *T. infestans* (Ti1) is n = 1,049; it shares 505 metabolites in common with Ti2 and 481 with Ti3. 258 metabolites are common among the three replicates. (*) is for the average number and standard deviation of sample size among triplicates and (**) is for those parameters concerning common metabolites between replicate pairs.

Data were heterogeneous among replicates within triatomine species (Figure 2). The level of variation was somewhat similar for *T. infestans* and *P. megistus* (Figures 2A and C), which was two to four times that of *R. prolixus* (Figure 2B). About half of the metabolites detected were common to two replicates, and a quarter was common to three of them (except in *R. prolixus*, where the proportion of common metabolites among triplicates reached ∼50% because of the lower variation among replicates). Again, the highest level of metabolite consistency among replicates was found in *R. prolixus*, as demonstrated by its higher metabolite conservation among pairs and lower standard deviation (Figure 2B). Thus, the metabolites common among replicates varied between ∼20% and ∼50% of the sample size per replicate among species.

**Figure 3:**
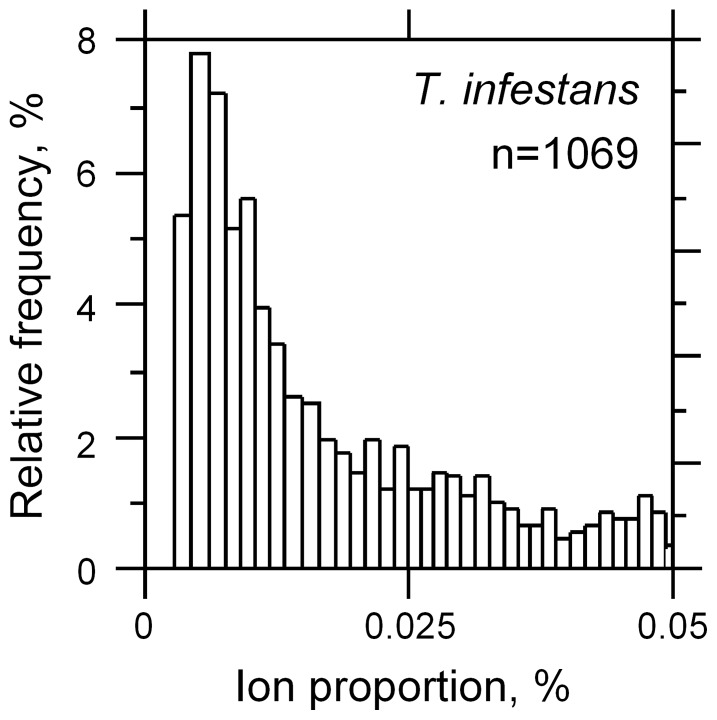
Representation of relative metabolite distribution. Most metabolites were found at very low rates (below 0.025%). The distribution extends above 0.05, up to 14%, but was not shown for clarity.

We found that despite their bimodal distribution according to their *m/z* values (Figure 1), metabolites from feces displayed a Poisson distribution according to their relative frequency (%) after normalization, i.e., the great majority of metabolites detected were found in small amounts (Figure 3). A similar situation was found in all three species (data not shown), which makes it difficult to make a decision concerning the specificity of a given metabolite in this class in light of the low level of statistical consistency (only three replicates).

To compare the metabolic repertoire of each triatomine species, we determined the correlation coefficients of average metabolite rates over three replicates between pairs of triatomine species. We found that the correlation coefficients for *T. infestans* vs. *R. prolixus*, *T. infestans* vs. *P. megistus* and *R. prolixus* vs. *P. megistus* were 0.88, 0.53 and 0.68, respectively. The higher level of correlation between *T. infestans* vs. *R. prolixus* suggested a greater similarity between the fecal metabolomes of these species. In contrast, the similarities of metabolite profiles were low for *T. infestans* vs. *P. megistus* and moderate for *R. prolixus* vs. *P. megistus*, suggesting a larger metabolic distance between *P. megistus* and *T. infestans* or *R. prolixus* than between *T. infestans* and *R. prolixus*.

**Figure 4:**
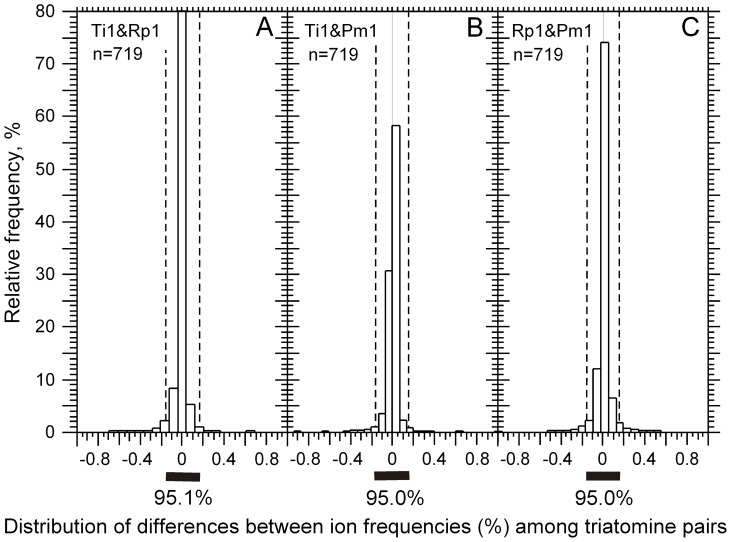
Distributions of metabolite rate differences. Three examples are given for the distributions of these differences among feces of different triatomine species, i.e., *T. infestans* vs. *R. prolixus* (A), *T. infestans* vs. *P. megistus* (B) and *R. prolixus* vs. *P. megistus* (C). The histograms focus on the significant part of the samples in terms of representativeness, but the values were found in a larger interval. In all panels, ∼95% of pair differences are found between −0.15 and +0.15 (n = 2,086).

**Figure 5:**
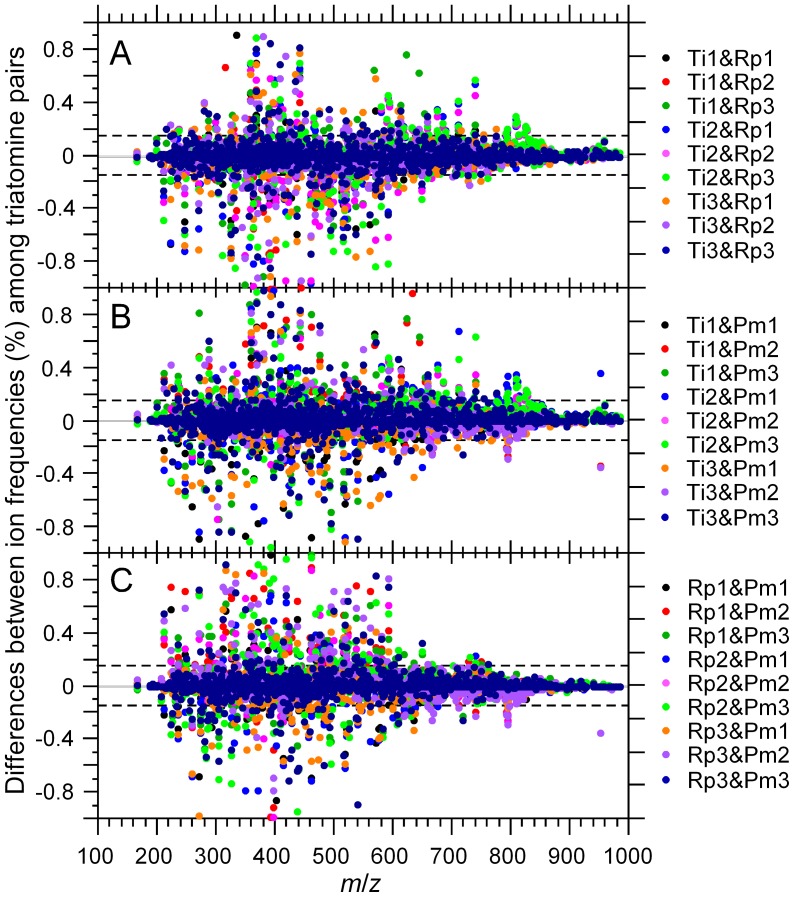
Differences in ion frequencies among triatomine replicates. Plots are given for all replicate combinations considering the following triatomine pairs: *T. infestans* vs. *R. prolixus* (A), *T. infestans* vs. *P. megistus* (B) and *R. prolixus* vs. *P. megistus* (C). Dots between dashed lines are for the metabolites with small differences among pairs of triatomine species. Dots outside the dashed lines are for the metabolites displaying large differences among pairs of triatomine species (at *p*≤0.05). For plotting convenience, the scale of the *y* axis has been limited to the interval −1 to +1. Some pairs exist outside this range (data not shown).

We found that the difference between the metabolite rates of a pair of replicates was a normal distribution centered on zero. The normality of metabolite rate differences among replicates of different species (Figure 4), such as in the case of *T. infestans* vs. *R. prolixus* (Figure 4A), *T. infestans* vs. *P. megistus* (Figure 4B) and *R. prolixus* vs. *P. megistus* (Figure 4C), was also verified. Because of the data normalization, we verified that the sums of differences were zero in all cases. However, their standard deviations were different (data not shown). Interestingly, the thresholds corresponding to the 95% confidence interval remained at −0.15 and 0.15 across the various combinations of replicate pairs. This shows that the rates of metabolites from the uniform core are monotonous across replicate pairs. Thus, it is the metabolites from the variable core that explain the standard deviation of the differences in the distributions of Figure 4. The distributions of Figure 4 are important for the rational classification between metabolites with both small and large fluctuations among triatomine species. Because it is reasonable to consider that metabolites with large variations may affect the physiology of *T. cruzi*, we set out to classify the metabolites by their variation between triatomine species. As explained in the Materials and Methods section, because the metabolite rate differences follow a normal distribution, the objective criterion of classification was a *p*-value smaller than α = 0.05 (5%). Because 95% of metabolite pairs are between −0.15 and 0.15 (Figure 4), we can set these values (−0.15 and 0.15) as the 5% threshold (α) for the classification (two-tailed t-tests) of metabolites with small (−0.15≤X≤0.15) or large variation ranges (X<−0.15 or X>0.15). The plots of Figure 5 show the distributions of metabolite rate differences according to their *m*/*z* value for all combinations of replicate pairs among the three triatomine species. The metabolites from the uniform cores, which showed small differences between species pairs, are between the −0.15 and 0.15 thresholds, and those from the variable core, which have a larger difference than expected in 95% of the cases, are outside these boundaries. The two-tail interval includes metabolites that are present at different levels in any two of the species being compared or are present in one of the species while absent in the other. It is also interesting to note that the metabolites of the variable core with the largest rate differences were found in a narrow *m*/*z* range, between 250 and 400, which matches the left mode of the *m*/*z* distribution when the scale extends from 250 to 1,000. The dots on the *x* axis (Figure 5) are not false positives; this situation occurs when a metabolite is present in one species but not in the other two and when its rate difference is larger than |0.15|. Thus, when a metabolite is absent in a pair of triatomines, it appears on *x* axis for that pair (data not shown).

Given the large number of frequency pairs (>1,000) analyzed in each replicate, the observed normal distribution can be considered representative of that of the whole population. Thus, we assume that the two-tailed threshold is represented by the dashed lines at the −0.15 and 0.15 values, which delimit 95% of the sample size. Therefore, when a metabolite is observed at relative frequencies differing by more than |0.15| in two triatomine species, its higher frequency in one of the two species must be considered out of the normal range at *p*≤5%, i.e., it is associated with some degree of correlative biological function (see Discussion).

**Figure 6:**
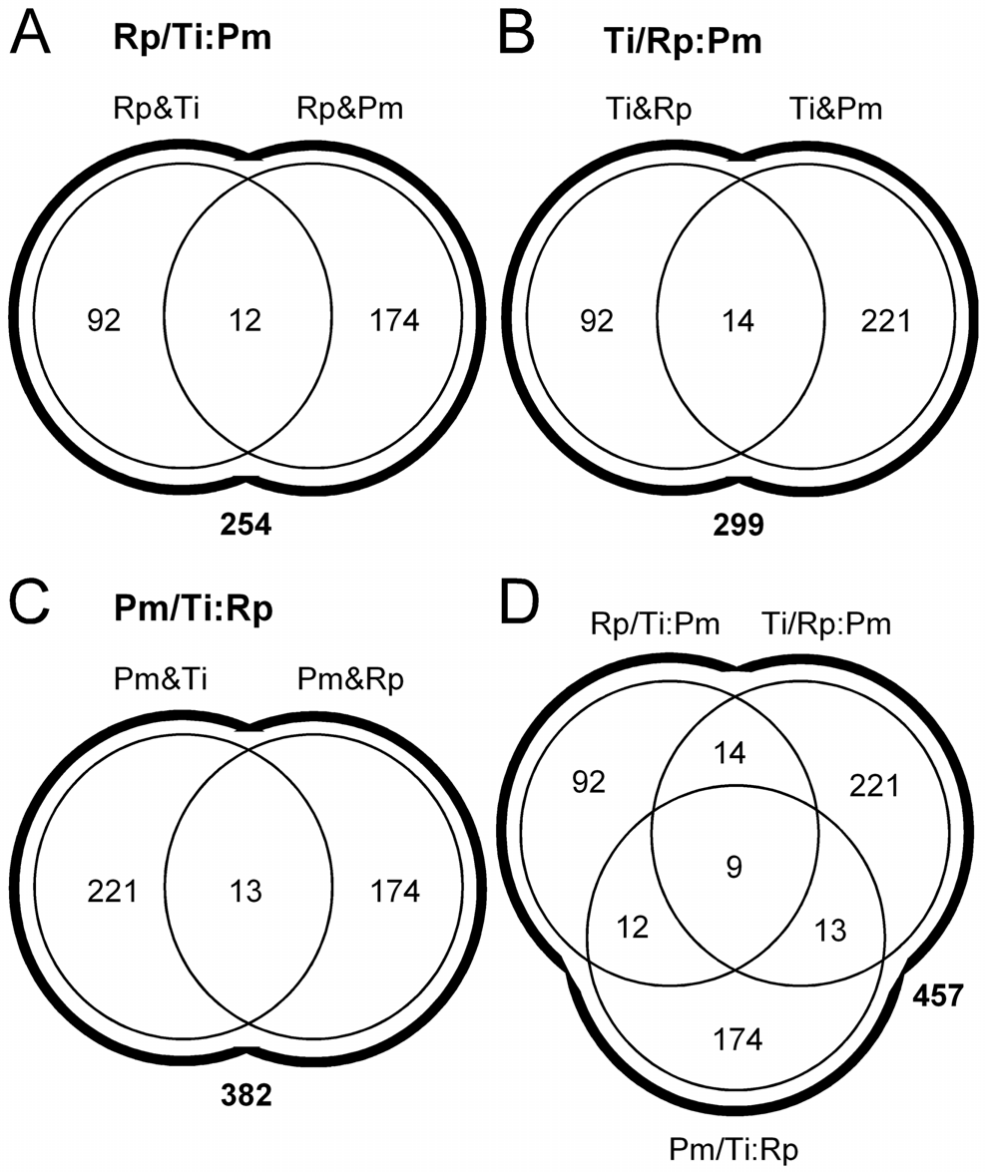
Boolean operations on metabolite rate differences. Venn diagrams are given for all replicate combinations considering the following triatomine comparisons: *R. prolixus* vs. *T. infestans* AND *P. megistus* (A), *T. infestans* vs. *R. prolixus* AND *P. megistus* (B), *P. megistus* vs. *T. infestans* AND *R. prolixus* (C), and all comparisons above (D).

The numbers of metabolites from the variable core found when comparing *T. infestans* to *R. prolixus*, *T. infestans* to *P. megistus,* and *R. prolixus* to *P. megistus* were 92, 221, and 174, respectively. The distribution of these metabolites among triatomine pairs can be represented by Venn diagrams, as shown in Figure 6. It is important to note that the metabolites from the variable core found when comparing one species to another may not be present when comparing that species to the third. These more complex situations can be explored using Boolean operations (OR and AND) when discussing the metabolite differences between triatomine pairs. To identify metabolites from the variable core in one species compared to the other two, the conditions set above for one species in comparison to another must hold for the two pairs involving the species considered (whatever the replicate combination considered, out of the 27 possible; 3 pairs of 3 triatomine species with 3 replicates each). Thus, the Boolean operations that we applied for all replicate pairs were (i) ((*R. prolixus* AND *T. infestans*) OR (*R. prolixus* AND *P. megistus*)) (Figure 6A), ((*T. infestans* AND *R. prolixus*) OR (*T. infestans* AND *P. megistus*)) (Figure 6B), ((*P. megistus* AND *T. infestans*) OR (*P. megistus* AND *R. prolixus*)) (Figure 6C), which corresponded to 254, 299, and 382 metabolites associated with the variable core, respectively, (ii) ((*R. prolixus* AND *T. infestans*) AND (*R. prolixus* AND *P. megistus*)) (Figure 6A), ((*T. infestans* AND *R. prolixus*) AND (*T. infestans* AND *P. megistus*)) (Figure 6B), ((*P. megistus* AND *T. infestans*) AND (*P. megistus* AND *R. prolixus*)) (Figure 6C), which corresponded to 12 (174+92–254), 14 (221+92–299), and 13 (221+174–382) metabolites, respectively, (iii) (*T. infestans* OR *R. prolixus* OR *P. megistus*), which corresponded to 457 (92+174+221-[12+14+13–9]) metabolites (Figure 6D) and (iv) (*T. infestans* AND *R. prolixus* AND *P. megistus*), which corresponded to 9 (457−92−174−221+12+14+13) metabolites (Figure 6D).

The numbers 254, 299, 382 (metabolites from (i)) are smaller than the sum of 92+174 = 266, 92+221 = 313, and 221+174 = 395, respectively, simply because the OR operator will count a metabolite specific to one species only once, which means that the cases just outlined account for 12, 14, and 13 metabolites, respectively, and these match the results found with the operator AND. Taking the test ((*T. infestans* AND *R. prolixus*) AND (*T. infestans* AND *P. megistus*)) as an example (Figure 6B), one can observe that the comparison between *R. prolixus* and *P. megistus* is not taken into account because of the comparison by pairs. Thus, some additional variable metabolites could be revealed by introducing the pair *R. prolixus* and *P. megistus*. This is effectively what we observed because the number of different metabolites classified as part of the variable core considering the union of the three species is not 487 (92+174+221), but instead is 457, which is the sum of the variable metabolites (92+174+221 = 487) less the variable metabolites common to a species pair, i.e., 30 = 12+14+13–9, because 39−30 = 9 metabolites are not common to all species when considering them together rather than by pairs, i.e., 487−30 = 457. This indicates that the intersection of the variable cores of the three species (Figure 6D), i.e., 9 metabolites, represents the metabolites that are present in at least one of the three replicates of a species and whose differences are larger than |0.15| when compared to any replicates of the two other species.

The statistics in Figure 6 show that, considering the whole metabolite cohort of this study (n = 2,086), the uniform core comprises 78% (1,628 metabolites), and the variable core comprises 22% (457 metabolites). Interestingly, a consequence of the analysis presented in Figure 6 is that different distribution profiles corresponding to the metabolites of the variable core were observed among triatomine species, which may indicate some species specificity in the quantitative features of the digestive tract among triatomines. However, larger replicate numbers would be necessary to give this observation any statistical consistency.

### The uniform core metabolome of the triatomine intestinal tract

**Figure 7:**
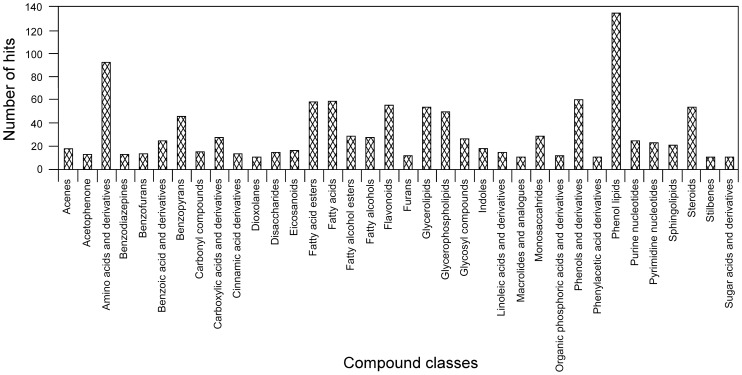
Metabolic classes in the uniform core. Frequency is given in number of hits per metabolic category in the uniform core. Only metabolic classes with 10 or more hits are displayed.

Through comparison to the Human Metabolome Database, we were able to annotate 1,051 metabolites, which represent 64.5% of the total uniform core metabolome (Table S2) with an average annotation redundancy of 3.8. However, the number of synonymous annotations varied a widely between 0 and 99, with 50% (523) non-redundant annotations and 90% under 10 synonymous annotations per entry. To avoid any ambiguity, we characterized the metabolites with non-synonymous annotations and found that they were distributed among many different metabolic classes (Figure 7). By comparing the frequency of a metabolic class to the average over all classes, we considered the classes with values larger than the average to be the most representative. Some of these classes were the following: amino acids and derivatives (6.0%), benzoic acid and derivatives (1.6%), benzopyrans (3.0%), carbonyl compounds (1.0%), carboxylic acids and derivatives (1.8%), cinnamic acid derivatives (0.9%), monosaccharides (1.8%), disaccharides (0.9%), eicosanoids (1.1%), fatty acids (3.8%), fatty acid esters (3.8%), fatty alcohols (1.8%), fatty alcohol esters (1.8%), prenol lipids (8.8%), phenols and derivatives (3.9%), purines and derivatives (1.6%), pyrimidines and derivatives (1.5%), flavonoids (3.6%), glycerolipids (3.5%), glycerophospholipids (3.2%), glycosyl compounds (1.7%), linoleic acid and derivatives (0.9%), sphingolipids (1.3%), steroids and derivatives (3.5%), acenes (1.1%), indoles (1.1%), and benzofurans (0.9%), among others (Table S6).

### The variable core metabolome of the triatomine intestinal tract

**Figure 8:**
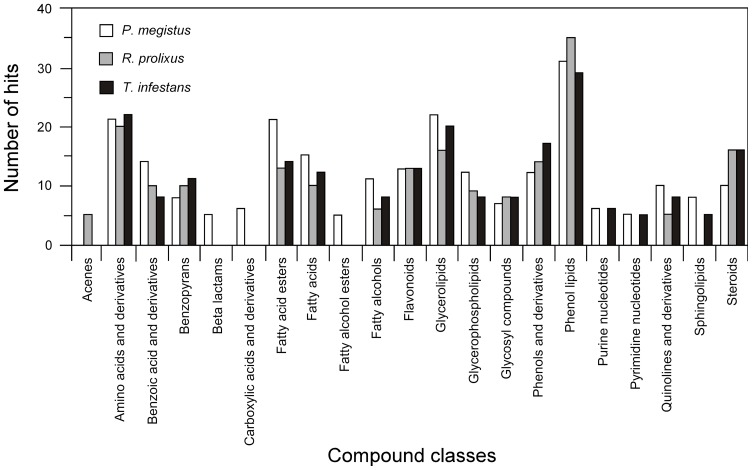
Metabolic classes in the variable core. Frequency is given in number of hits per metabolic category in the following comparisons: *P. megistus* vs. *T. infestans* and *R. prolixus* (white bars), *R. prolixus* vs. *T. infestans* and *P. megistus* (gray bars), and *T. infestans* vs. *R. prolixus* and *P. megistus* (black bars). Only metabolic classes with 5 or more hits are displayed.

By comparison to the Human Metabolome Database, we were able to annotate 445 metabolites out of the 935 that represented the combined variable core of the three triatomine species studied (382 for *P. megistus*, 254 for *R. prolixus,* and 299 for *Triatoma infestans*). This represents 47.6% of the total variable core metabolome (Tables S3, S4 and S5). The annotations of the variable core metabolites showed that although some metabolites correlated with the different species considered, many of the most significant pathways were relatively conserved among these species, with prenol lipids, glycerolipids, amino acids, fatty acid esters, steroids, phenols and fatty acids their major determinants. With this in mind and using the data generated by HMDB, we manually grouped the metabolites from the variable cores in metabolic classes to determine the most significant metabolic pathways. By comparing the individual metabolic class frequencies to the average frequency over all classes we determined the most representative metabolic classes in the variable core. These classes were prenol lipids (Pm/Rp&Ti: 7.8%, Rp/Pm&Ti: 11.4%, Ti/Pm&Rp: 8.1%), glycerolipids (Pm/Rp&Ti: 5.5%, Rp/Pm&Ti: 5.2%, Ti/Pm&Rp: 5.6%), amino acids (Pm/Rp&Ti: 5.3%, Rp/Pm&Ti: 6.5%, Ti/Pm&Rp: 6.1%), fatty acid esters (Pm/Rp&Ti: 5.3%, Rp/Pm&Ti: 4.2%, Ti/Pm&Rp: 3.9%), fatty acids (Pm/Rp&Ti: 3.8%, Rp/Pm&Ti: 3.2%, Ti/Pm&Rp: 3.4%), benzoic acid and derivatives (Pm/Rp&Ti: 3.5%, Rp/Pm&Ti: 3.2%, Ti/Pm&Rp: 2.2%), flavonoids (Pm/Rp&Ti: 3.3%, Rp/Pm&Ti: 4.2%, Ti/Pm&Rp: 3.6%), glycerophospholipids (Pm/Rp&Ti: 3.0%, Rp/Pm&Ti: 2.9%, Ti/Pm&Rp: 2.2%), phenols (Pm/Rp&Ti: 3.0%, Rp/Pm&Ti: 4.5%, Ti/Pm&Rp: 4.7%), fatty alcohols (Pm/Rp&Ti: 2.8%, Rp/Pm&Ti: 1.9%, Ti/Pm&Rp: 2.2%), quinolones (Pm/Rp&Ti: 2.5%, Rp/Pm&Ti: 1.6%, Ti/Pm&Rp: 2.2%), steroids (Pm/Rp&Ti: 2.5%, Rp/Pm&Ti: 5.2%, Ti/Pm&Rp: 4.5%), and others (Figure 8 and Table S6). Careful analysis of this dataset shows that only a few of the most important categories of the variable core were different from those of the uniform core (azoles, quinolines, hydroxyl acids, indoles; Table S6). Comparing the uniform and variable cores, we found correlation coefficients of 0.98, 0.93, 0.94 (uniform core versus Pm/Ti&Rp, Rp/Ti&Pm, and Ti/Pm&Rp, respectively). These high levels of correlation (>0.90) between the uniform and variable cores confirm that the metabolic categories that are above average in the uniform core are also those that are the most variable in the triatomines studied. Therefore, these categories are likely the ones that contribute most to the modulation of *T. cruzi*'s biology during its life cycle in the triatomine gut.

## Discussion

DI-FT-ICR-MS is a technique that enables the precise quantitative characterization of metabolites in a biological sample by data accumulation, allowing the fast and accurate derivation of the metabolic profile associated with a sample [24], [25]. However, one drawback to this method is that the qualitative characterization of each molecular specimen relies on the comparison of its molecular weight with preexisting information stored in a database. As is common with such approaches, a lack of information in databases precludes the full characterization of a set of samples. In this report, we studied the fecal metabolome of three species of triatomines, the insect vectors of *T. cruzi* and, hence, Chagas disease. To obtain putative identities of the metabolic features detected, we searched the Human Metabolome Database (HMDB) due to the lack of an appropriate database for triatomine samples. The HMDB has wide metabolic coverage, currently containing information on more than 40,000 metabolites, which prompted us to use this database for our studies. However, it is important to note that an appreciable proportion of the molecules detected in our study did not have any hits in the HMDB and therefore remained unidentified. Thus, full characterization of the metabolic features described herein will require the expansion of metabolic coverage by the HMDB as well as the development of databases directed toward the analysis of insect samples.

In agreement with uniform practice in biology, we analyzed three replicates of fecal samples per triatomine species. This sample size does not allow variance stabilization, which usually occurs when n≅100. Therefore, some metabolites that were considered part of the uniform core may actually belong to the variable core. In contrast, metabolites from the variable core are unlikely to belong to the uniform core, simply because if they can be considered statistically variable with only three repetitions, the level of certainty can only increase with repetition number. In fact, statistical confidence in the determination of whether a metabolite is part of the uniform or variable core can be obtained with three replicates because the threshold associated with this choice is based on a much larger sample size of metabolites (n = 2,086), which warrants statistical consistency according to *p*≤0.05.

Limitations notwithstanding, we used DI-FT-ICR-MS to characterize the fecal metabolome of three species of triatomines and to identify subsets of metabolites that are either uniform to all species or variable among them. In doing so, we found that the metabolites conserved among the three species (those of the uniform core) pertained to multiple metabolic classes, with fatty acids, steroids, glycerolipids, amino acids, sugars, and nucleotides being widely represented. As previously discussed, given that parasite differentiation (metacyclogenesis) takes place in the triatomine gut, the chemical environment encountered by *T. cruzi* is likely to affect this process. As such, the molecules described here as uniform to all species of triatomines (Table S1) may play key roles in the life cycle of the parasite.

We showed that lipids and fatty acids are the most abundant metabolite classes in the feces of all triatomines studied. Lipids play a fundamental role in the biological cycle of *T. cruzi*. Lipid extracts from metacyclogenic intestinal preparations were shown to induce significant differentiation of epimastigotes into infective metacyclic trypomastigotes [26]. The authors also showed that the total fraction of blood lipids represented by lysophosphatidylcholine, phosphatidylethanolamine, phosphatidylinositol, phosphatidylcholine, triacylglycerol and sphingomyelin is quickly degraded into free fatty acids in the triatomine intestinal tract and is incorporated into epimastigotes. It is known that free fatty acids are imported into the trypanosomatid cell via an ABC transporter, a superfamily of ATP-binding cassette transporters [27]. In addition, the rate of metacyclogenesis induction by the total lipid fraction was shown to be about half of that obtained by a total intestinal extract and similar to that of free fatty acids or oleic acid alone (used as a representative of the free fatty acid fraction). Thus, oleic acid alone is able to mimic half the rate of metacyclogenesis induced by the whole intestinal extract and promote trypomastigote viability and integrity. Another collateral effect observed by these authors was the biosynthesis of phosphatidylcholine and diacylglycerol by epimastigotes, as well as the activation of protein kinase C, as a consequence of free fatty acid accumulation due to the digestion of blood phospholipids by an intestinal phospholipase. This is an expected consequence of the inositol phosphate/diacylglycerol signaling pathway that has been described in *T. cruzi* [28]. In this sense, the triatomine digestive process could be linked to metacyclogenesis via protein kinase C activation through diacylglycerol biosynthesis. The feedback regulation of diacylglycerol biosynthesis occurred through the correlated synthesis of phosphatidylcholine. Interestingly, the signaling pathway of metacyclogenesis induced by free fatty acids is different from that induced by cAMP [26], [29]. In fact, *T. cruzi* epimastigotes are induced to differentiate into metacyclic trypomastigotes by increased cAMP levels that result from the addition of catecholamines (epinephrine), which are tyrosine derivatives that bind to a subclass of G-protein-coupled receptors [30].

Phospholipids are complex molecules that are essential for intracellular signaling and membrane integrity [31] but are also precursors for several other very important lipid molecules including sphingolipids, ceramides and glycosylphosphatidylinositol anchors [32]. Certain glycosylphosphatidylinositol-anchored proteins play a special role in the virulence of protozoan pathogens as adhesins and/or variable epitopes that are used to evade the immune system, such as the variable surface glycoprotein in *T. brucei* and *T. cruzi* [33], [34]. Myo-inositol (inositol) is an essential nutrient that is used for building phosphatidylinositol and its derivatives in eukaryotes. As a consequence, protozoan pathogens must be able to acquire inositol to proliferate and infect their hosts. Phosphatidylinositol is also the precursor for a wide variety of membrane-bound and non-membrane-bound phosphorylated inositol signal transduction molecules [35]. *T. cruzi* has at least two different myo-inositol transporters based on a biochemical analysis of import activities [36]. The central role of phospholipids in *T. cruzi* metabolism has been demonstrated by the antiprotozoal activity of several phospholipid analogs [37].

In addition to fatty acids and phospholipids, steroids are another important group of molecules based on their relative metabolite proportion in triatomine feces. The main biological activity associated with this metabolic group is hormonal. For instance, ecdysone, a steroid produced by prothoracic glands, was shown to be involved in the epithelial cell organization of the *R. prolixus* midgut and the dysregulation of the neuroendocrine system induced by azadirachtin (an insect growth inhibitor that blocks ecdysis). Interestingly, the epithelial midgut alteration is correlated with parasite death, indicating that the epithelial extracellular membranes are involved in the establishment and development of *T. cruzi* in the gut of this vector [38]. The beneficial effect of steroids on *T. cruzi* has been shown in the chronic stage of Chagas disease in humans [39]. In addition, *T. cruzi* itself is able to produce androgens and estrogens when incubated in the presence of steroid precursors, which suggests the presence of active parasite steroidogenic enzymes and increases the significance of the observation of steroid metabolites in triatomine feces [40], [41].

Glycerolipids are formed through the linkage of fatty acids to glycerol by ester bonds; many of these lipids have biological activities, such as diacylglycerol, platelet-activating factor, choline and ethanolamine, for example [42]. In addition to their role in energy metabolism, glycerolipids have roles in cellular signaling for many biological processes [43], [44]. Of interest in this study, trypanosomatids synthesize lipids from acetyl-CoA and glycerolipids from glycerol through the mevalonate pathway [41]. First, triacylglycerol and glycerophospholipids are synthesized from phosphatidic acid. Basically, phosphatidic acid is dephosphorylated by a phosphatidate phosphatase. The resulting diacylglycerol can be directly acetylated by an acyltransferase to form a triacylglycerol or can react with cytidine-diphosphate-choline (CDP-choline) or CDP-ethanolamine to form phosphatidylcholine or phosphatidylethanolamine, respectively. Ethanolamine phosphate cytidylyltransferase and choline phosphate cytidylyltransferase homologs were identified in trypanosomatids [45]. Ethanolamine, phosphatidylcholine and sphingomyelin are classes of phospholipids that are abundant in cell membranes. Another example of a phospholipid with an essential role in the biology of *T. cruzi* is lysophosphatidylcholine. This compound, present in saliva and feces of *R. prolixus*, is a powerful chemoattractant for inflammatory cells at the site of the insect bite, creating a concentrated population of cells for parasite infection. In addition, lysophosphatidylcholine increases intracellular calcium concentrations in macrophages, ultimately enhancing parasite invasion. Finally, lysophosphatidylcholine inhibits nitric oxide production in macrophages stimulated by *T. cruzi* and thus interferes with the immune system of the vertebrate host [46].

Because the chemical profile of metabolites in the triatomine intestine may affect parasite development, it is important to understand how a certain species of triatomine may tune the ecological niche of *T. cruzi*. Indeed, many metabolic classes found in our study, such as prenol lipids, amino acids, glycerolipids, steroids, phenols, fatty acids and derivatives (esters and alcohols), benzoic acid and derivatives, flavonoids, glycerophospholipids, benzopyrans, and quinolones were enriched in the triatomine species studied (Tables S3, S4, and S5). These data show that in addition to factors previously shown (or hypothesized) to affect the interaction of *T. cruzi* and its vector (such as hemolysins, lectins and pigments) [14], there are many chemical determinants of the intestinal environment that may be specifically tuned based on the species of triatomine involved, and this may also affect the vector-parasite interaction. The concept of *metabolic niche* in parasitic protozoa has been extensively discussed [47]. Given the availability of an assortment of metabolites within the host, it is not surprising that some pathways were abandoned in obligate parasites such as trypanosomatids as opposed to opportunistic parasites. The gut is an environment with reduced oxygen availability, which implies anaerobic fermentation of glucose and amino acid carbon sources. *T. cruzi* exhibits a specific optimization to allow the metabolism of histidine to glutamate. Glutamate can then be converted to α-ketoglutarate, a precursor of the citric acid cycle, which correlates with the occurrence of histidine as the predominant free amino acid in *R. prolixus* feces [48].

An important group of fatty acid derivatives that may be involved in vector specificity is the eicosanoids, which are oxygenated metabolites of polyunsaturated fatty acids (PUFAs) [49]. PUFAs cannot be synthesized *de novo* by most animals or protists and must be obtained from dietary plant products [50]. Eicosanoids are a family of lipid mediators that participate in a wide range of biological activities in animals [51]. In insects, eicosanoids are mainly synthesized from arachidonic acid released from cell membrane phospholipids via phospholipase A_2_ activation. Arachidonic acid is subsequently metabolized via the following three pathways: (i) the cyclooxygenase (COX) pathway, forming prostaglandins, thromboxanes or prostacyclins; (ii) the various lipoxygenase (LOX) pathways, forming leukotrienes, lipoxins, hepoxilins, hydro(pero)xy and hydroxy fatty acids; and (iii) the cytochrome P-450 (CYP450) pathways, forming epoxy derivatives [52]. In insects, including *R. prolixus*, eicosanoids mediate specific cell actions, including phagocytosis, microaggregation, nodulation, hemocyte migration, hemocyte spreading and the release of prophenoloxidase in reaction to bacterial and protozoan challenges [6], [15]. According to insect models [53], the chemical components of infecting microorganisms, such as lipopolysaccharide, stimulate a number of intracellular transduction systems, including those responsible for upregulation of eicosanoid biosynthesis by phospholipase A_2_ activation. Arachidonic acid released from the plasma membrane is subsequently converted into prostaglandin E_2_ (PGE_2_) or other eicosanoids. Prostaglandins are exported from the cell by specific transporter proteins. The prostaglandins can interact with receptors on the exporting cell or on neighboring cells [52]. Moreover, it has been demonstrated that *T. cruzi* has access to the arachidonic acid pathway through the Old Yellow Enzyme [54]. *T. cruzi* also synthesizes eicosanoids, preferentially thromboxane A_2_ (TXA_2_). Parasite-derived TXA_2_ alone is sufficient to mediate disease progression in the human host and seems to be essential in modulating the host response to the parasite to ensure the orientation of disease pathogenesis toward the chronic phase and promote the long-term sustainability of the host-parasite relationship [55]. Other eicosanoids released by *T. cruzi* may also contribute to vertebrate parasite infection [56]. Therefore, the abundance and types of lipids available to *T. cruzi* during vector infection will affect its ability to synthesize eicosanoids, which can have a direct impact on both the parasite itself and on the vector host. This in turn can affect the outcome of the vector-parasite interaction. In support of this concept, it has recently been shown that the production of eicosanoids during *T. cruzi* infections of mammalian hosts has an effect on parasite burden, and this may also be true during triatomine infections [57].

In humans, dietary preferences and nutritional composition have been shown to influence gut microbial metabolism and, correlatively, health [58]. Similarly, a number of B vitamins, namely nicotinamide, thiamin, pyridoxine, riboflavin, p-aminobenzoic acid and biotin, were shown to be essential to *R. prolixus* molting and were associated with symbionts [59]. Choline and folic acid are B vitamins that are essential to *T. cruzi* growth in minimal medium [60]. In contrast, p-aminobenzoic acid, biotin and pyridoxine were not essential to *T. cruzi*. Folates are conjugated pterines that contain p-aminobenzoic acid and L-glutamates connected to the methyl group at position 6 of the pteridine ring system. The activity of extracellular folic acid is regulated by a folic acid synthase. The products were identified as pterin-6-aldehyde and p-aminobenzoylglutamic acid. *T. cruzi* is an auxotroph for folate and pteridine, which are imported through permeases. The purpose of folate metabolism is to supply reduced folates for the conversion of deoxyuridine 5′-phosphate (dUMP) to deoxythymidine 5′-phosphate (dTMP) and form thymidine for DNA replication [61]. Dihydrofolate reductase (DHFR) catalyzes the two-step reduction of folate to tetrahydrofolate, which is then transformed to N5, N10-methylene tetrahydrofolate and is used by thymidylate synthase (TS) as a methyl donor and reducing agent in the conversion of dUMP to dTMP.

In trypanosomatids, reduced pteridines are essential for a number of important cellular functions. After salvage, pteridine precursors are reduced to their respective biologically active tetrahydro forms by the pteridine reductase PTR1. The DHFR-TS used by trypanosomatids can exclusively reduce folic acid; the reductase PTR1 shows a much broader range of activity, catalyzing successive reductions of conjugated (folate) and unconjugated (biopterin) pterins. If DHFR-TS is inhibited, PTR1 can be overexpressed, allowing for significant reduction of the necessary amount of folates to ensure parasite survival. Because of this compensatory mechanism, the development of any drug targeting the folate pathway in trypanosomatids should consider the inhibition of both DHFR and PTR1 [62].

Many factors may be critical to promoting metabolic diversification among triatomine species, as described above. One determinant of the gut chemical composition is the intestinal microbiota. A recent study showed that the chemical composition of mouse feces is highly disturbed by killing the intestinal microbiota [22]. The gut microbiota of triatomines was recently studied by our group [12]. The results showed that the composition of bacterial microbiota varies among triatomine species but is conserved among the individuals of one species, regardless of their geographic origin. In contrast, insect vector competence may vary with geographic location and may affect *T. cruzi* epidemiology. The relative regularity of microbiota in the triatomine guts regardless of the geographic location of a given vector species is not surprising, because the host determinants were shown to be essential factors of microbiota composition [63]. In addition, the vast majority of the bacterial community was composed of endosymbiotic species as determined by DGGE [12]. In that sense, one should consider bacterial microbiota as a rather constant part of the vector system that may affect *T. cruzi in situ*. Another potential determinant of the metabolic changes observed here is the digestive process of each triatomine species. Although knowledge of triatomine physiology is fragmented among insect vector species, *R. prolixus* digestion has been studied for many years, and the activities of several cysteine and/or aspartic proteinases have been identified in the posterior midgut [64], [65], [66]. More recent studies have demonstrated the presence of genes encoding cathepsins B and L in the midgut of *R. prolixus* and *T. infestans*, respectively [67], [68]. In addition to differences in digestive enzymes in the gut, in *P. megistus*, *T. infestans* and *R. prolixus* defecation is stimulated by blood ingestion, which immediately induces the discharge of a high amount of feces after feeding [14], [69]. Therefore, differences in the timing and volume of this excretion between triatomine species may affect the metabolic composition of feces. In reality, all of the factors mentioned above will contribute to the chemical composition of the insect gut, and dissecting their individual contributions will be a challenge worth undertaking.

In summary, we have shown that triatomine feces constitute a rich and varied chemical medium whose metabolite constituents are likely to affect *T. cruzi* development and infectivity. In addition to the potential effects of the uniform core of metabolites as the primary determinants of the success of the host-parasite relationship between triatomines and *T. cruzi*, variable metabolites may act as secondary determinants and are likely critical to the course of the host-parasite relationship. These secondary determinants may play their roles through direct actions as well as synergistic or antagonistic effects of multiple metabolites whose proportions may vary according to the insect host. It is well known that different *T. cruzi* strains do not have the same developmental success in different triatomine species and different ecological conditions; *T. cruzi* virulence varies according to the geographic location. All three species tested here are good vectors for *T. cruzi*. Thus, comparing the metabolome outlined here to that of triatomines with poor *T. cruzi* transmission efficiency would most likely aid in identifying factors involved in the development of *T. cruzi*.

Interestingly, compounds like eicosanoids, which may play a role in metacyclogenesis, varied quantitatively among triatomine species in our study. Because the developmental success of a *T. cruzi* strain in its invertebrate host may depend on the triatomine species and the intestinal microbiota depends on the triatomine species, we suggest that a tripartite relationship is critical to the interactions between triatomines and *T. cruzi*. In addition, *T. cruzi* can also affect the microbiota composition upon triatomine infection [70], which, in turn, may affect the metabolic composition of triatomine feces, with significant feedback consequences on the parasite. Altogether, these observations suggest that a specific homeostatic system controlling the epidemiology of Chagas disease may exist. Therefore, it is necessary to determine how the three biological components of the triatomine-*T. cruzi* host-parasite interaction may affect the developmental success of the parasite. As such, this work is the first attempt to understand the complexity of the fecal metabolome in triatomines as a path for further investigations of triatomine vector competence for specific *T. cruzi* strains. Our work has opened many avenues for investigations concerning the role of individual metabolites in the interactions among *T. cruzi,* its triatomine vectors and the vertebrate host. Knowledge of these interactions is likely to generate new ways to understand the factors influencing parasite proliferation and spread as well as methods to control Chagas disease.

## Supporting Information

Methods S1 **Statistical analysis.**
(DOCX)

Table S1 **Raw Direct Infusion Fourier Transform Ion Cyclotron Resonance Mass Spectrometry data.**
(XLSX)

Table S2 **Putative metabolite identity assignments for the uniform core using the Human Metabolome Database.**
(XLSX)

Table S3 **Putative metabolite identity assignments for the variable core (Pm vs Ti-Rp) using the Human Metabolome Database.**
(XLSX)

Table S4 **Putative metabolite identity assignments for the variable core (Rp vs Ti-Pm) using the Human Metabolome Database.**
(XLSX)

Table S5 **Putative metabolite identity assignments for the variable core (Ti vs Pm-Rp) using the Human Metabolome Database.**
(XLSX)

Table S6 **Metabolic classes in the uniform and variable cores of T. infestans (Ti), R. prolixus (Rp) and P. megistus (Pm).**
(XLSX)
