# Supplementary material for: Metabolic Signatures of Triatomine Vectors of Trypanosoma cruzi Unveiled by Metabolomics
Source: PLoS One. 2013 Oct 30;8(10):e77283. doi: 10.1371/journal.pone.0077283 (PMC3813737; doi:10.1371/journal.pone.0077283)
Supplement: Methods S1 — Statistical analysis. (DOCX) [file pone.0077283.s001.docx]

**Methods S1*.*** Statistical analysis. We first normalized the absolute *m/z* peak intensities to the total intensity of each sample. Thus, the sum of relative frequencies of metabolites was always 100%, and their difference among replicate pairs (three per species) was always zero. Since the distribution of replicate pair differences was a normal centered on zero for each case, we looked for the two tail threshold values between which 95% of data were found (-0.15 and +0.15). Metabolites affected by small and large rate differences between triatomine pairs are linked by a probability function such that their difference of relative frequency in different triatomine species follow a normal distribution centered on zero. Thus, asking whether a given metabolite has a trend to be found at a large rate in a triatomine species compared to another one or not is the same as asking for the null hypothesis that this metabolite is at equal frequencies in the two triatomine species considered. If the metabolite is found at a large rate in one species of the pair, the difference of its frequency among triatomines of this pair will be high. The probability of this event is especially low as the difference of metabolite rates among the pair is large. When the difference of metabolite rates among the pair is larger than a threshold, statistical methods recommend to reject the null hypothesis of equality of metabolite frequencies in two species, i.e., to consider the larger rate of the metabolite in one species as significant under p<5%. This threshold is generally considered as an acceptable compromise between type I error (the rejection of the null hypothesis when it is actually correct) and type II error (the acceptance of the null hypothesis when it is actually incorrect). The decision that a metabolite may be found at a larger rate in one triatomine species than in another is a two-tailed test because no qualitative differences exist between both tails since the difference between a metabolite in two triatomine species is commutative. The metabolites whose differences among replicate pairs of a given triatomine species were inside the range between the two tail thresholds (-0.15 and +0.15) were considered belonging to the uniform core. Consequently, the metabolites whose differences among replicate pairs were outside the range between the two tail thresholds were considered belonging to the variable core at *p*<5%. The test of hypothesis described herein allows us to transform a quantitative distribution in a qualitative statement (TRUE or FALSE) according to the question of whether a metabolite belongs to the uniform or variable core. Thus, the question of whether a metabolite belongs to the uniform or variable core can be easily extended to the comparison of replicates among two or three triatomine species by combining Boolean operators OR (union) and AND (intersection). This can be done because 95% of metabolite differences among replicate pairs were found between the same two tail thresholds (-0.15 and +0.15) for all three triatomine species.

For a metabolite in one species to be considered belonging to the variable core, the modulus (unsigned value) of its difference in all replicate pairs of that species with those of the two other triatomine species under comparison must be larger than 0.15 except if, for some reason, it has not been observed in one replicate (this is an undefined situation where no conclusion can be drawn). The 2,086 metabolites covered by this study were not present in each replicate of all species and there is not enough data to consider the absence of a metabolite in a replicate as statistically consistent. Thus, we counted the differences of a metabolite larger than |0.15| in all combinations of the three species comparing one to the second OR to the third. More formally, to report on the statistical significance of metabolite rate differences among pairs of triatomine species (referred to as Rp&Ti, Rp&Pm and Pm&Ti below), we considered the condition X < -0.15 OR X > 0.15, assigning TRUE calls when the rate difference of a metabolite (X) in a replicate pair was larger than |0.15| and FALSE calls when -0.15 < X < 0.15 except in the case where X = 0, which we considered as undefined (UNDEF). However, an UNDEF call in one pair of replicates cannot invalidate the decision in another pair involving the same two triatomine species. Thus, the general goal is to make a decision (Y) of whether the metabolite must be considered as part of the variable core or not. Y was considered TRUE (the metabolite belongs to the variable core) when X = TRUE OR X = 0 over (AND) all pairwise combinations among replicates. In other words, according to this rule, Y can only be TRUE if X = 0 OR X < -0.15 OR X > 0.15, but not if -0.15 < X < 0.15 for any pair combination of replicates between two species of the three triatomines. Thus, if one considers the case Rp&Ti, a metabolite will be considered belonging to the variable core if Y_Rp1xTi1_ AND Y_Rp1xTi2_ AND Y_RpnxTin_ is TRUE, where Y_RpnxTin_ ∈ {1 < n < ξ}, with n being the replicate under consideration and ξ being the maximum number of replicates in this study, i.e., 3.

When counting the differences of a metabolite larger than |0.15| in all combinations of the three species comparing one to the second OR to the third, as explained above, the OR operator can be substituted by AND. Such substitution would result in a much smaller set (intersection), given the restriction introduced by AND, which supposes the condition to be TRUE in ALL considered cases. However, it has been demonstrated that the infectivity of a *T. cruzi* strain (and thus its potential risk for humans) varies among vector species. Consequently, a metabolite showing a significant difference in a replicate pair, but not in another, could still be relevant from an epidemiological standpoint. In fact, it is the profile associated to metabolites of the variable core that may be relevant, and not specific metabolites. Thus, because of its potential vector specificity, the *union* of Y=TRUE cases over X metabolites in one triatomine species is valuable from an epidemiological standpoint. By contrast, the intersection of all combinations across the three triatomine species obtained with the AND operator would give the metabolites that are specific to one species regarding the two others. Therefore, we implemented the OR and AND cases as follows: the statistics of Boolean tests can be easily represented by Venn diagrams by counting occurrences (1 for TRUE and 0 for FALSE) according to the tests just described. This methodology allows giving account of the metabolite distribution among the uniform and variable cores of triatomine species in a very simple way and without ambiguity.
